# Nitrite of Amyl in Ague

**Published:** 1879-12

**Authors:** 


### NITRITE OF AMYL IN AGUE.

Dr. W. E. Saunders, of Indore, calls attention to the value of nitrite of amyl in ague, and records a number of cases in which advantage has been derived from its use. The drug itself, he remarks, is inexpensive, and goes a long way.

He now uses *amyl nitrite* mixed with an equal part of *oil of coriander* to render it less volatile, and at the same time to cover its odor. He regards it as the most powerful diaphoretic he has seen, and he uses it in all cases of fever to produce diaphoresis.

The following is one of his cases: Mr. F. C. came for treatment about 7 P. M., in the cold stage of ague. Two minims of nitrite of amyl were administered. Sweating came on in seven minutes. He lay down for half an hour to get cool, and then walked home well. He, next morning, took a dose of quinine, and has had but one attack of fever without the cold stage since. Previous to this he had fever every day for one month, during which he took large doses of quinine.

Dr. Saunders observes that he does not mean to say that quinine should not be used in these cases, for there is ample proof that it tends to check the return of the attacks, and removes to some extent the septic condition of the blood, induced by the malarial poison, and this more especially if small doses of opium are combined with it. In no case did the amyl fail to remove the attack in about one-third the usual time, and in most cases the fever did not return. His method of administration is this: Four drops of the above mixture, or two of amyl, are poured on a small piece of lint, which is given into the hands of the patient, and he is told to inhale it freely. He soon becomes flushed, and both his pulse and respiration are much accelerated. When he feels warm all over, the inhalation is discontinued, as the symptoms continue to increase for some time afterwards. A profuse perspiration now sets in, which speedily ends the attack. In some cases, however, the cold stage passes off without any hot or sweating stage.—*Indian Medical Gazette in The Practitioner.*

